# Random Material Property Fields of 3D Concrete Microstructures Based on CT Image Reconstruction

**DOI:** 10.3390/ma14061423

**Published:** 2021-03-15

**Authors:** George Stefanou, Dimitrios Savvas, Panagiotis Metsis

**Affiliations:** Department of Civil Engineering, Institute of Structural Analysis & Dynamics of Structures, Aristotle University of Thessaloniki, 54124 Thessaloniki, Greece; dimitriosavvas@yahoo.gr (D.S.); panos@metsis.gr (P.M.)

**Keywords:** concrete, CT images, reconstruction, 3D FE models, homogenization, mesoscale random fields

## Abstract

The purpose of this paper is to determine the random spatially varying elastic properties of concrete at various scales taking into account its highly heterogeneous microstructure. The reconstruction of concrete microstructure is based on computed tomography (CT) images of a cubic concrete specimen. The variability of the local volume fraction of the constituents (pores, cement paste and aggregates) is quantified and mesoscale random fields of the elasticity tensor are computed from a number of statistical volume elements obtained by applying the moving window method on the specimen along with computational homogenization. Based on the statistical characteristics of the mesoscale random fields, it is possible to assess the effect of randomness in microstructure on the mechanical behavior of concrete.

## 1. Introduction

The macroscopic mechanical properties of heterogeneous materials consisting of multiple phases such as particle-reinforced composites and concrete are significantly influenced by their underlying microstructure and can be determined through numerical homogenization. The application of homogenization methods in the analysis of multi-phase materials offers an efficient alternative over direct simulation of the microstructure in terms of required computational resources. The key issue in homogenization methods is the linking of micromechanical characteristics with the random variation of material properties at higher scales, which is usually established using Hill’s macro-homogeneity condition [1]. In this framework, it is necessary to identify a representative volume element (RVE) usually through computational convergence schemes with respect to specific apparent properties [2]. In contrast to the RVE, which is characterized by deterministic material properties, the spatial variation of the elasticity tensor at lower scales is quantified by random fields computed on mesoscale models—statistical volume elements (SVEs) [3,4].

The highly heterogeneous microstructure of concrete is responsible for its complex mechanical behavior. Therefore a wide variety of methods have been developed for the accurate reconstruction of concrete microstructure mostly based on image processing and random fields. The geometrical characteristics of aggregates are statistically analyzed in [5] by processing 2D images of concrete obtained from X-ray computed tomography (CT). A recent review on the use of micro-CT images to investigate the relation between microstructural features and properties of cement-based materials is provided in [6] showing the important role of threshold determination on phase identification. Two non-Gaussian random field models are developed in [7] for simulating the geometry of star-like inclusions in heterogeneous materials. For illustration, the models are calibrated to the geometrical features of a population of aggregates used in concrete. Huang and Peng [8] present a multiscale computational framework for generating concrete microstructures by concurrently coupling stationary Gaussian and fractional Brownian random fields, used to simulate coarse and fine aggregates, respectively.

The mechanical properties of concrete are determined using computational homogenization in a number of publications. Constantinides and Ulm [9] confirm the existence of two types of calcium-silicate-hydrate (C-S-H) in cement-based materials, and examine the effect of the two phases on the Young’s modulus and Poisson ratio of this kind of material by means of nanoindentation tests. The effective properties of cement pastes are computed using a homogenization model based on the elastic properties and volume fractions of the two phases. Wriggers and Moftah [10] generate 3D concrete microstructures taking into consideration the random aggregate morphology at the mesoscale. Then the finite element (FE) method is used for the homogenization of concrete. In [11], 2D and 3D homogenization and fracture analysis of concrete are performed based on micro-CT images and Monte Carlo simulation. Tal and Fish [12] determine a realistic RVE of concrete, which represents its random microstructure. A probabilistic upscaling procedure for modeling the mesoscale elastic properties of concrete at the level of SVE is provided by Dubey and Noshadravan [13]. To the authors’ knowledge, the random spatial variability of the mechanical properties of concrete at various scales has not been thoroughly investigated yet.

The purpose of the present paper is to provide a complete probabilistic description of the spatial variation of the elastic properties of concrete taking into account its highly heterogeneous microstructure, which is reconstructed using CT images of a cubic concrete specimen. The variability of local volume fraction of the constituents (pores, cement paste and aggregates) is quantified first. Mesoscale random fields of the elasticity tensor are computed subsequently from a number of SVEs obtained by applying the moving window method on the concrete specimen along with computational homogenization. Based on the statistical features (empirical distribution and correlation functions) of the random fields, it is possible to assess the effect of randomness in microstructure on the mechanical behavior of concrete.

The paper is organized as follows: In Section 2, the process of reconstructing concrete microstructure based on CT images is described. Section 3 presents the framework of computational homogenization used to determine the random spatially varying apparent elasticity tensor of 3D concrete microstructures. The numerical results obtained using the proposed approach are discussed in the next section followed by some concluding remarks in Section 5.

## 2. Reconstruction of Concrete Microstructure Based on CT Images

### 2.1. CT Images

**Figure 1:**
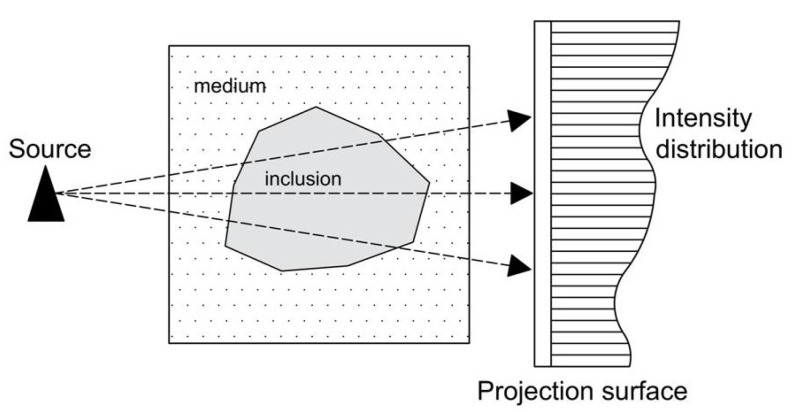
Intensity Distribution of an X-ray array.

Computed Tomography (CT) has its origins in the medical field and refers to a computerized X-ray imaging procedure in which a patient is struck by a narrow X-ray beam that rotates at a certain speed around the patient’s body. While the CT procedure has been applied mainly for medical purposes, the gradual increase in available CT equipment and the improvement of the cost factors have led to other utilizations, such as the exploration of the microstructure of heterogeneous materials [14]. When the X-ray beam passes through a sample, some of the X-ray radiation is absorbed and scattered, while the rest penetrates through the sample, as shown in Figure 1.

The absorbed or scattered radiation of a material can be measured in Hounsfield units (HU). HU is a quantitative scale that reflects the linear transformation of the original linear attenuation coefficient measurement (μ) so as the radiodensity of distilled water at standard pressure and temperature is defined as zero HU, while the radiodensity of air, at the same conditions, is defined as −1000 HU. The linear transformation used to convert the attenuation values μ to HU values on each pixel of the CT image is as follows:

(1) HU=μ×slope+intercept

where the values of the rescale slope and intercept parameter are specific to the CT scanner system used [15,16]. Their values can be obtained from the extracted DICOM (Digital Imaging and Communications in Medicine) files.

### 2.2. Reconstruction of Concrete Microstructure

**Figure 2:**
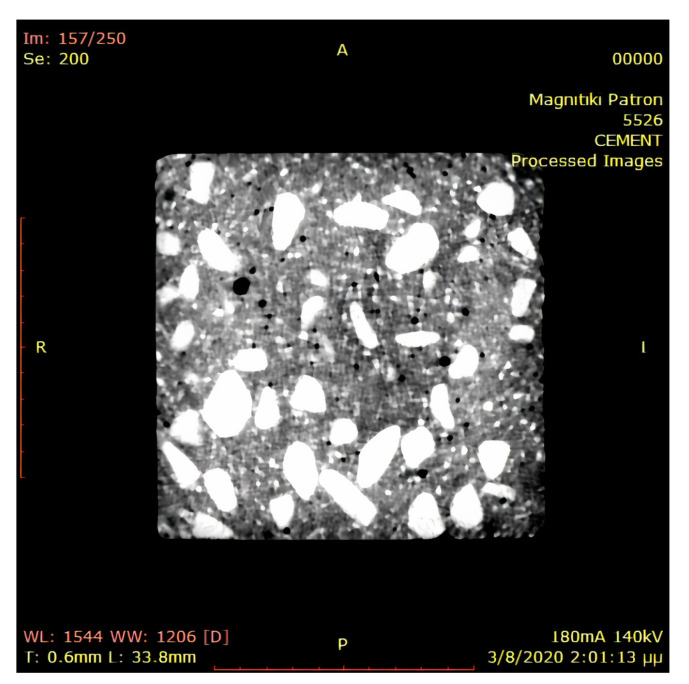
A 2D computed tomography (CT) image of concrete.

The reconstruction of concrete microstructure is based on data obtained from the CT scan. The specimen is cut in 2D slices (images) with their corresponding resolution (Figure 2). Each pixel carries, beside its geometric properties, the attenuation value μ that is converted to the respective HU value using Equation (1). Then, by utilizing voxels (the generalization of pixels on a regularly spaced, three-dimensional grid), the voxel-based FE method is employed. Using the moving window technique [17], random mesoscale models or SVEs are created, which are discretized by a structured FE mesh based on voxels geometry.

In this work, the medical CT scan was conducted at the laboratory of Magnitiki Patron S.A. [18], which has provided all the DICOM data for the reconstruction of concrete microstructure. The equipment used was a LightSpeed VCT of GE Medical Systems Hellas (Athens, Greece) with peak potential and current applied to the X-ray tube equal to 140 kV and 180 mA, respectively. The axial resolution of the scan was fixed at 0.625 mm resulting in 211 total slices, while the pixel resolution of the 2D image in each slice plane is 0.4883×0.4883 mm${}^{2}$, resulting in 281×281 pixels. Note that the dimensions of the scanned cubic concrete specimen was approximately 136.724×136.724×131.25 mm${}^{3}$. For the visualization of the image data the RadiAnt DICOM viewer software [19] has been used.

## 3. Computational Homogenization

A fundamental assumption in the homogenization process is the statistical homogeneity of the heterogeneous medium [20]. This means that, at any material point of the medium all statistical properties of the state variables are the same and thus identification of the RVE is possible. In case of composites with periodic or nearly periodic geometry, the RVE is explicitly defined [21] whereas in case of spatial randomness, the RVE needs to be determined using computational methods [3,22,23,24,25,26,27].

A basic principle for the existence of the RVE is separation of scales as defined below [1]:

(2) $$d\ll L\ll L_{macro}$$

where the microscale parameter *d* denotes a characteristic size of the fillers, e.g., the average dimension of aggregates, the mesoscale parameter *L* denotes the size of the volume element and the macroscale parameter $L_{macro}$ denotes the characteristic length over which the macroscopic loading varies in space. Note that, in the case of complete scale separation, $L_{macro}$ denotes the size of the macroscopic homogeneous medium.

**Figure 3:**
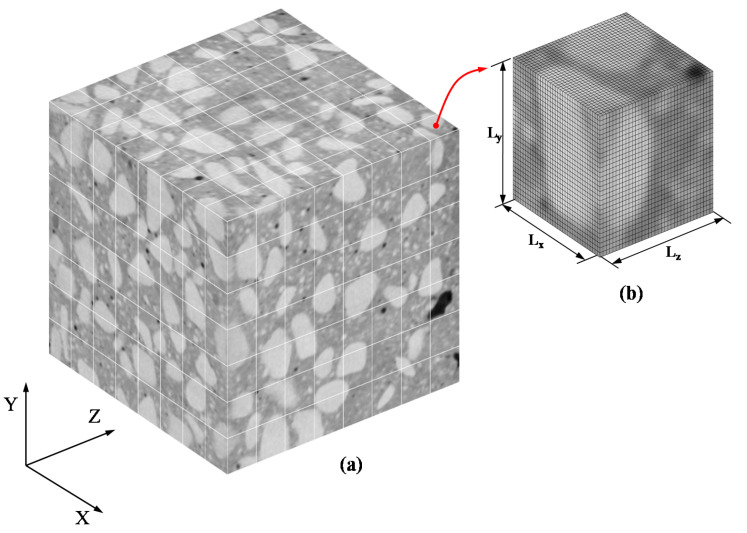
(**a**) Schematic of 3D moving window technique on a cubic concrete specimen reconstructed using 2D CT images, (**b**) Detail of the voxel based finite element (FE) mesh of a specific statistical volume element (SVE) model of dimensions $L_{x}\times L_{y}\times L_{z}$.

The statistical characteristics of the apparent elasticity tensor of concrete are computed by analyzing a set of random mesoscale models or SVEs. These are obtained by applying the moving window technique on a cubic concrete specimen reconstructed based on a set of 2D CT images (see Section 2). Figure 3 depicts the cubic concrete specimen segmented into a large number of non-overlapping SVEs along with a detail of the voxel based FE mesh of a specific SVE. Each SVE model is characterized by the scale factor δ=L/d, which is a non-dimensional parameter with $\delta \in \left[1,\infty \right]$. The length scale of the SVE is always smaller than the one of the corresponding RVE. For each positioning of the moving window, the apparent material properties are computed through homogenization. The obtained mesoscale properties and corresponding window centres are used to form the related random field model, as shown in Section 4.

### Computation of the Random Apparent Elasticity Tensor of 3D Concrete Microstructures

The stress and strain fields on a mesoscale realization of concrete $B_{\delta }\left(\omega \right)$ can be written as:

(3) $$\sigma \left(\omega ,x\right)=\bar{\sigma }+\sigma^{\prime }\left(\omega ,x\right)\;\;,\;\;\varepsilon \left(\omega ,x\right)=\bar{\varepsilon }+\varepsilon^{\prime }\left(\omega ,x\right)$$

where $\sigma^{\prime }$ and $\varepsilon^{\prime }$ are the zero-mean fluctuations and $\bar{\sigma }$ and $\bar{\varepsilon }$ are the means of the stress and strain tensors. At some point X of the macro-continuum the above means can be computed as volume averages over $B_{\delta }\left(\omega \right)$ [1]:

(4) $$\bar{\sigma }\left(\omega ,X\right)=\frac{1}{V_{\delta }}\int_{B_{\delta }\left(\omega \right)}\sigma \left(\omega ,x\right)\;dV_{\delta },\;\;\bar{\varepsilon }\left(\omega ,X\right)=\frac{1}{V_{\delta }}\int_{B_{\delta }\left(\omega \right)}\varepsilon \left(\omega ,x\right)\;dV_{\delta }$$

where $V_{\delta }=\int_{B_{\delta }\left(\omega \right)}dV_{\delta }$ and ω denotes randomness of a quantity.

Moreover, the strain energy can be calculated as:

(5) $$\bar{U}=\frac{1}{2V_{\delta }}\int_{B_{\delta }\left(\omega \right)}\sigma \left(\omega ,x\right):\varepsilon \left(\omega ,x\right)\;dV_{\delta }=\frac{1}{2}\bar{\sigma :\varepsilon }=\frac{1}{2}\bar{\sigma }:\bar{\varepsilon }+\frac{1}{2}\bar{\sigma^{\prime }:\varepsilon^{\prime }}$$

It is noted that for an unbounded space domain the fluctuation terms in Equation (5) become too small and thus they can be neglected according to Hill’s condition:

(6) $$\bar{\sigma :\varepsilon }=\bar{\sigma }:\bar{\varepsilon }$$

Hill’s condition is also valid for a finite size of the domain, provided that the following constraint is satisfied [28]:

(7) $$\int_{\partial B_{\delta }}\left(t-\bar{\sigma }\cdot n\right)\cdot \left(u-\bar{\varepsilon }\cdot x\right)\;dS=0$$

where *t*, *u* are the traction and displacement vectors, respectively, and *n* is the unit normal vector on surface dS. Equation (7) is satisfied by appropriate boundary conditions (e.g., uniform kinematic or static, orthogonal-mixed and periodic). In this study, computational homogenization is performed by applying uniform kinematic boundary conditions on the SVEs (see Miehe and Koch [29]).

In general the 3D elasticity tensor $\bar{C}$, which is of the 4th order, consists of $3^{4}=81$ components. These are reduced to 21 independent components due to symmetries in stress and strain tensors. Thus, the macroscopic linear constitutive relation for an anisotropic material is as follows:

(8) $$\bar{\sigma }=\bar{C}\bar{\varepsilon }\Rightarrow \left[\begin{array}{c} {\bar{\sigma }}_{11}\\ {\bar{\sigma }}_{22}\\ {\bar{\sigma }}_{33}\\ {\bar{\sigma }}_{12}\\ {\bar{\sigma }}_{23}\\ {\bar{\sigma }}_{31} \end{array}\right]=\left[\begin{array}{cccccc} {\bar{C}}_{1111} & \; & & \; & & \mathrm{sym}\\ {\bar{C}}_{2211} & {\bar{C}}_{2222} & \; & & \; & \\ {\bar{C}}_{3311} & {\bar{C}}_{3322} & {\bar{C}}_{3333} & \; & & \\ {\bar{C}}_{1211} & {\bar{C}}_{1222} & {\bar{C}}_{1233} & {\bar{C}}_{1212} & \; & \\ {\bar{C}}_{2311} & {\bar{C}}_{2322} & {\bar{C}}_{2333} & {\bar{C}}_{2312} & {\bar{C}}_{2323} & \\ {\bar{C}}_{3111} & {\bar{C}}_{3122} & {\bar{C}}_{3133} & {\bar{C}}_{3112} & {\bar{C}}_{3123} & {\bar{C}}_{3131} \end{array}\right]\left[\begin{array}{c} {\bar{\varepsilon }}_{11}\\ {\bar{\varepsilon }}_{22}\\ {\bar{\varepsilon }}_{33}\\ {\bar{\varepsilon }}_{12}\\ {\bar{\varepsilon }}_{23}\\ {\bar{\varepsilon }}_{31} \end{array}\right]$$

The unknown macroscopic elasticity components ${\bar{C}}_{ijkl}$, with i,j,k,l=1,2,3, are equal to the macroscopic stresses ${\bar{\sigma }}_{ij}$, which are derived by the solution of six independent kinematic uniform boundary value problems (BVPs). In each BVP the displacement boundary conditions are obtained by a prescribed uniform unit strain tensor as shown below:

(9) $$\bar{\varepsilon }=\left\{\left[\begin{array}{c} {\bar{\varepsilon }}_{11}=1\\ 0\\ 0\\ 0\\ 0\\ 0 \end{array}\right],\left[\begin{array}{c} 0\\ {\bar{\varepsilon }}_{22}=1\\ 0\\ 0\\ 0\\ 0 \end{array}\right],\left[\begin{array}{c} 0\\ 0\\ {\bar{\varepsilon }}_{33}=1\\ 0\\ 0\\ 0 \end{array}\right],\left[\begin{array}{c} 0\\ 0\\ 0\\ {\bar{\varepsilon }}_{12}=1\\ 0\\ 0 \end{array}\right],\left[\begin{array}{c} 0\\ 0\\ 0\\ 0\\ {\bar{\varepsilon }}_{23}=1\\ 0 \end{array}\right],\left[\begin{array}{c} 0\\ 0\\ 0\\ 0\\ 0\\ {\bar{\varepsilon }}_{31}=1 \end{array}\right]\right\}$$

The imposed displacements on the boundary nodes of each SVE are calculated through the prescribed uniform strain tensor using the following equation:

(10) $$u_{b}=D_{b}^{T}\bar{\varepsilon }$$

where the geometric matrix $D_{b}$ is calculated for a boundary node *b* based on its coordinates:

(11) $$D_{b}=\frac{1}{2}\left[\begin{array}{ccc} 2x_{b} & 0 & 0\\ 0 & 2y_{b} & 0\\ 0 & 0 & 2z_{b}\\ y_{b} & x_{b} & 0\\ 0 & z_{b} & y_{b}\\ z_{b} & 0 & x_{b} \end{array}\right]\;\;\mathrm{with}\;\;\left(x_{b},y_{b},z_{b}\right)\in x$$

The BVP which has to be solved is as follows:

(12) $$\left[\begin{array}{cc} K_{ii} & K_{ib}\\ K_{bi} & K_{bb} \end{array}\right]\left[\begin{array}{c} u_{i}\\ u_{b} \end{array}\right]=\left[\begin{array}{c} f_{i}\\ f_{b} \end{array}\right]$$

where the stiffness matrix K of the FE model is rearranged into four sub-matrices associated with interior nodes *i* and boundary nodes *b*. The macroscopic stress tensor is then calculated as a volume average by:

(13) $$\bar{\sigma }=\frac{1}{V}Df_{b}$$

where $f_{b}=K_{bb}u_{b}+K_{bi}u_{i}$ is the vector containing the computed reaction forces on the boundary nodes, $u_{i}=K_{ii}^{-1}\left(f_{i}-K_{ib}u_{b}\right)$ is the vector containing the calculated displacements of the interior nodes and $D=\left[D_{1}\;D_{2}\;\ldots \;D_{n_{b}}\right]$ with $n_{b}$ the total number of boundary nodes *b*.

## 4. Numerical Results and Discussion

In this section, numerical results concerning the determination of the random fields, the empirical distributions and the 3D spatial correlations of the components of the homogenized elasticity tensor of the 3D concrete specimen are presented for various scale factors δ examined. As shown in Figure 3, the size of each SVE corresponds to that of the selected 3D window in the moving window technique. Moreover, the moving window step in each direction has been selected equal to the size of the examined window so as non-overlapping SVEs are extracted. In the following parametric study the examined mesoscale sizes $\delta_{i}$ with *i* = 1, 2, 3, 4, correspond to different window sizes and thus different total numbers of SVEs (see Table 1).

In the context of FE simulation, the discretization of the SVEs into hexahedral solid elements is based on the voxels which are used to reconstruct the 3D geometry of the concrete specimen. Information about the pixels (e.g., location, spacing, attenuation values μ) and the slices (e.g., position, thickness) needed in order to construct the voxels and also to identify the constituent materials is included in the DICOM files provided by the CT scan system. In order to reduce the required computational cost, each hexahedral solid element in the FE mesh of the SVEs corresponds to 2×2×2 voxels. The material assigned to each integration point of an element depends on the HU value of the integration point which is calculated by interpolating the nodal HU values using the shape functions of the element.

**Figure 4:**
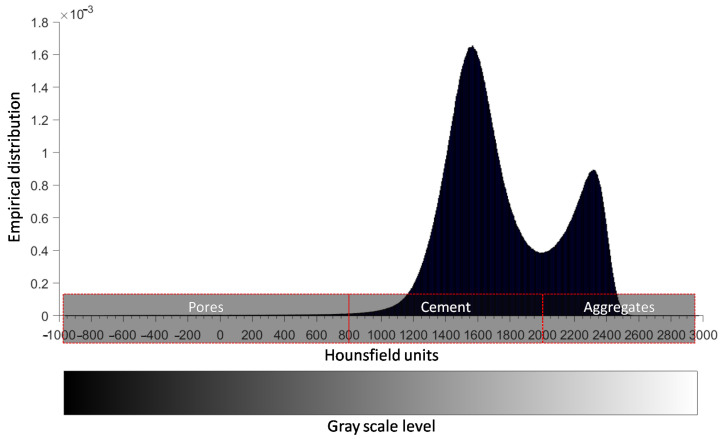
Empirical distribution of Hounsfield unit (HU) values derived from all CT images followed by the range of HU for concrete constituent materials.

Figure 4 depicts the histogram (empirical distribution) of the Hounsfield units (HU) calculated by Equation (1) with rescale slope and rescale intercept equal to 1 and −1024, respectively, for the CT equipment used. From this figure, the constituent materials (pores, cement and aggregates) can be distinguished by defining specific ranges for their HU values. Based on the observed peaks of the histogram and after careful processing of the CT images using RadiAnt DICOM viewer, the HU range can be selected as [−960, 800] for pores, [800, 2000] for cement and [2000, 2974] for aggregates. Figure 4 depicts also a gray scale colorbar related to the HU values of the constituent materials of concrete. The illustration of concrete specimen in Figure 3 is based on this color range.

**Figure 5:**
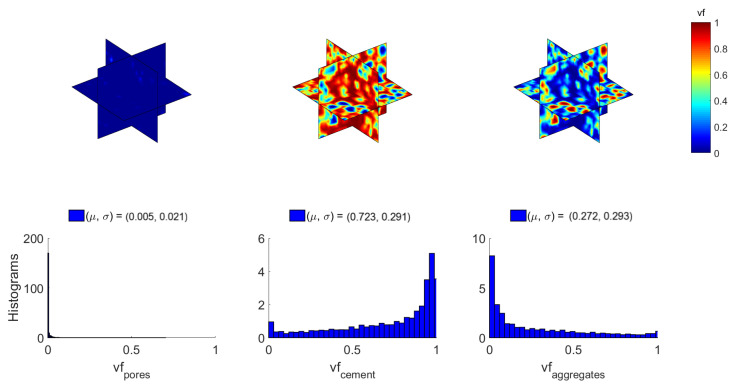
Random fields and histograms of volume fraction (vf) of concrete constituents for $\delta_{1}$.

**Figure 6:**
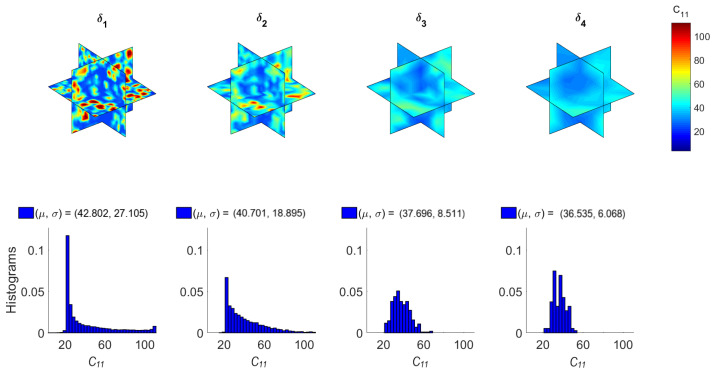
Random fields and histograms of ${\bar{C}}_{11}$.

**Figure 7:**
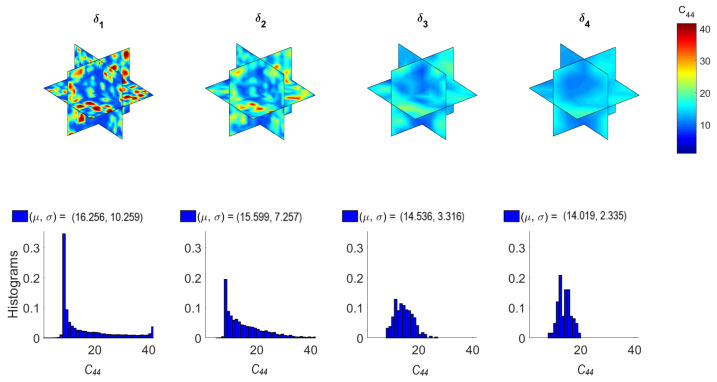
Random fields and histograms of ${\bar{C}}_{44}$.

Based on the aforementioned HU ranges, the local volume fraction (vf) variability of the constituents of concrete can be studied next. Figure 5 illustrates the computed 3D random fields along with the respective empirical distributions of the volume fraction of the constituent materials for $\delta_{1}$. The dispersion of aggregates within cement paste is not uniform and also, some air was trapped inside the paste during the mixture process. These facts are evident in Figure 5, where regions of material rich or poor in aggregates and pores can be observed. The mean vf of the constituents in the concrete specimen is 0.5% pores, 72.3% cement and 27.2% aggregates. For visualization purposes, Figure 5, Figure 6 and Figure 7 provide 2D contour plots of the random fields corresponding to the three orthogonal planes (XY, YZ and ZX) intersected at the center point of the cubic concrete specimen.

Figure 6 and Figure 7 illustrate the computed random fields and the empirical distributions of the apparent ${\bar{C}}_{11}$ (axial) and ${\bar{C}}_{44}$ (shear) stiffness with respect to $\delta_{i}$ (see Equation (8) with correspondence of subscripts: 1→11 and 4→12). These results have been obtained by implementing the homogenization method presented in Section 3 in each mesoscale size $\delta_{i}$. The constituent materials of concrete are considered linear elastic. Specifically, their Young’s modulus and Poisson ratio [E, v] are assumed as [20 GPa, 0.2] for cement and [100 GPa, 0.2] for aggregates. Note that pores can be modeled by setting their Young’s modulus equal to 0.2 GPa (1% of the Young’s modulus of cement) and Poisson ratio equal to 0.45 (almost incompressible material). From Figure 6 and Figure 7, it can be observed that random fields become less variable and histograms narrow as the scale factor δ increases. Specifically, the coefficient of variation (COV) reduces from 63% to 17% for both ${\bar{C}}_{11}$ and ${\bar{C}}_{44}$ as δ increases. However, it is deduced that morphological uncertainty can lead to significant random spatial variation of the mechanical properties of concrete.

**Figure 8:**
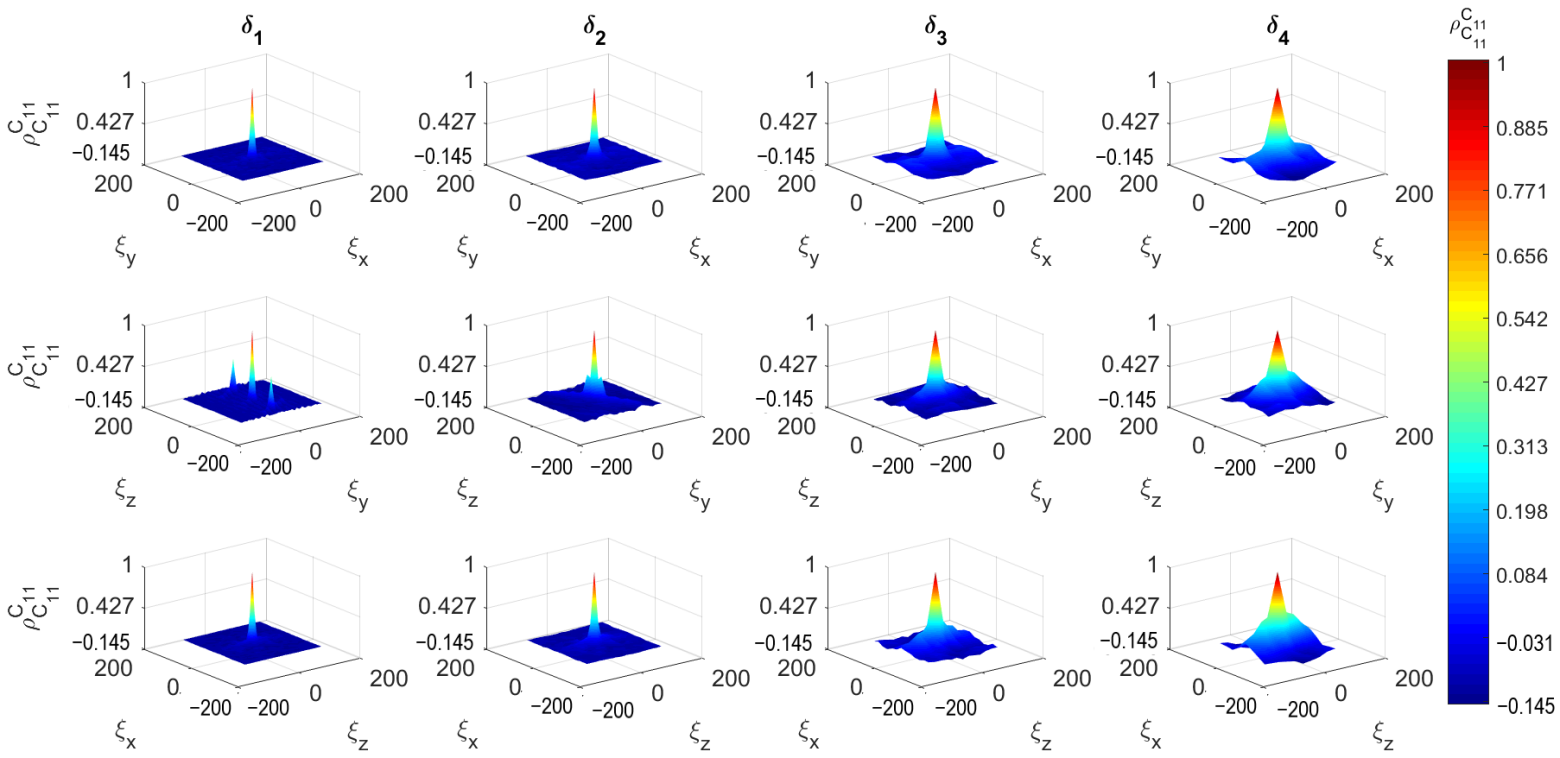
Auto-correlation function of ${\bar{C}}_{11}$.

**Figure 9:**
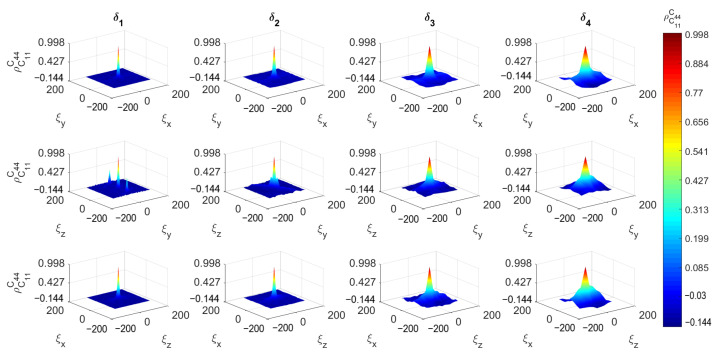
Cross-correlation function between ${\bar{C}}_{11}$ and ${\bar{C}}_{44}$.

**Figure 10:**
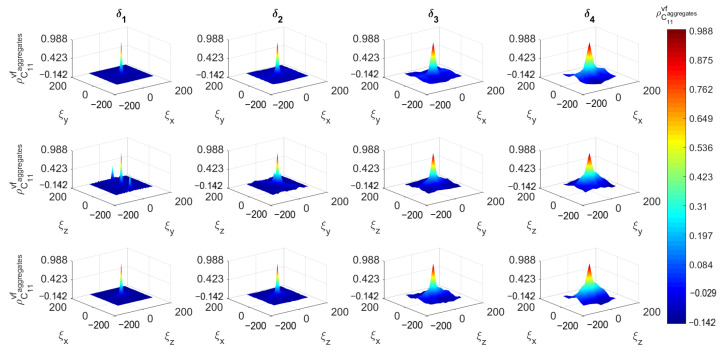
Cross-correlation function between ${\bar{C}}_{11}$ and vf of aggregates.

Figure 8, Figure 9 and Figure 10 depict the 3D spatial correlations of ${\hat{\rho }}_{{\bar{C}}_{11}}^{{\bar{C}}_{11}}$, ${\hat{\rho }}_{{\bar{C}}_{11}}^{{\bar{C}}_{44}}$ and ${\hat{\rho }}_{{\bar{C}}_{11}}^{{\mathrm{vf}}_{\mathrm{aggr}}}$, respectively, for increasing mesoscale size $\delta_{i}$. For visualization purposes, the aforementioned figures depict the spatial correlation values corresponding to the three orthogonal planes (XY, YZ and ZX) intersected at the center point of the cubic concrete specimen. The estimates of the spatial correlation functions ${\hat{\rho }}_{A}^{B}$ have been calculated for every lag $\left(\xi_{x},\;\xi_{y},\;\xi_{z}\right)$ according to the following formula:

(14) $$\begin{array}{c} \begin{array}{cc} {\hat{\rho }}_{A}^{B}\left(\xi_{x},\;\xi_{y},\;\xi_{z}\right) & =\frac{1}{n-1}\sum_{i=0}^{n_{wx}}\sum_{j=0}^{n_{wy}}\sum_{k=0}^{n_{wz}}\\ \; & \left(\frac{A\left(x^{i},\;y^{j},\;z^{k}\right)-{\hat{\mu }}_{A}}{{\hat{\sigma }}_{A}}\right)\left(\frac{B\left(x^{i}+\xi_{x},\;y^{j}+\xi_{y},\;z^{k}+\xi_{z}\right)-{\hat{\mu }}_{B}}{{\hat{\sigma }}_{B}}\right) \end{array}\;\\ -n_{wx}\Delta \xi_{x}\leq \xi_{x}\leq n_{wx}\Delta \xi_{x}\;,\;-n_{wy}\Delta \xi_{y}\leq \xi_{y}\leq n_{wy}\Delta \xi_{y}\;,\;-n_{wz}\Delta \xi_{z}\leq \xi_{z}\leq n_{wz}\Delta \xi_{z} \end{array}$$

with ${\hat{\rho }}_{A}^{B}$ denoting auto-correlations when A≡B, otherwise cross-correlations are defined. ${\hat{\mu }}_{A}$, ${\hat{\mu }}_{B}$ are the sample mean values while ${\hat{\sigma }}_{A}$, ${\hat{\sigma }}_{B}$ are the sample standard deviations of quantities *A*, *B*, respectively. The total number of SVEs is $n=n_{wx}{\dot{n}}_{wy}{\dot{n}}_{wz}$. It can be observed that the correlation length of all mesoscale random fields increases as the scale factor δ increases. In other words, the random fields become fully correlated for large δ [30]. The auto-correlations for lag $\left(\xi_{x}=0,\;\xi_{y}=0,\;\xi_{z}=0\right)$ are 1 and tend to zero for lag values $\left|\xi_{x}\right|>L_{x}$, $\left|\xi_{y}\right|>L_{y}$ and $\left|\xi_{z}\right|>L_{z}$. The cross-correlations have similar behavior with maximum value at zero lag less than 1. This can be attributed to the fact that the probability of SVEs to share common aggregates is decreased as the length of the vector ${\vec{\xi }}_{p}=\left[\xi_{x},\xi_{y},\xi_{z}\right]$ is increased.

**Figure 11:**
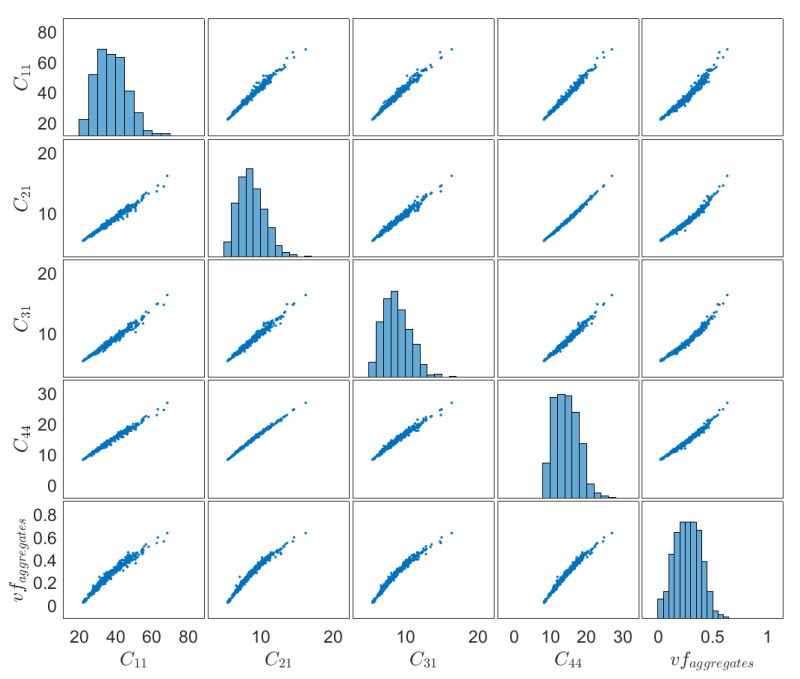
Scatter plots of elasticity components ${\bar{C}}_{11}$, ${\bar{C}}_{21}$, ${\bar{C}}_{31}$, ${\bar{C}}_{44}$ and vf of aggregates for $\delta_{3}$ (correspondence of subscripts: 1→11, 2→22, 3→33, 4→12).

Figure 11 presents scatter plots of elasticity components ${\bar{C}}_{11}$, ${\bar{C}}_{21}$, ${\bar{C}}_{31}$, ${\bar{C}}_{44}$ and vf of aggregates corresponding to data obtained in $\delta_{3}$. These plots show that there is a strong positive correlation among all elasticity components and also between the elasticity components and the vf of aggregates, which verifies the fact that morphological uncertainty in the microstructure of concrete can affect significantly its homogenized properties.

## 5. Conclusions

- In this paper, the random spatially varying apparent elasticity tensor of 3D concrete microstructures has been computed using CT images of a cubic specimen for the reconstruction of concrete microstructure and computational homogenization.
- A complete probabilistic description of the spatial variation of the elastic properties of concrete has been achieved through computation of the respective mesoscale random fields. In this way, it was possible to assess the effect of randomness in microstructure on the mechanical behavior of concrete based on the statistical characteristics (empirical distribution and correlation functions) of the random fields.
- A moderate to large variability of the local volume fraction of the constituents (pores, cement paste and aggregates) has been observed depending on the scale factor δ considered.
- This was reflected on the moderate to high COV of the apparent axial and shear stiffness, meaning that microstructural uncertainty can lead to substantial random spatial variation of the mechanical properties of concrete.
- The computed random material property fields can be used for the stochastic FE analysis of concrete structures.
- The consideration of the interfacial transition zone (ITZ) between cement paste and aggregates and the determination of random fracture properties could constitute the subject of future research.

## Figures and Tables

**Table 1:** Size of window and total number of SVEs corresponding to each $\delta_{i}$ examined.

$\delta_{i}$	$L_{x}\times L_{y}\times L_{z}$ (mm${}^{3}$)	No. SVEs
$\delta_{1}$	4.88×4.88×6.25	16,464
$\delta_{2}$	9.77×9.77×8.75	2940
$\delta_{3}$	19.53×19.53×18.75	343
$\delta_{4}$	27.34×27.34×26.25	125

## Data Availability

The data used in this study are available from the second author (D.S.) upon reasonable request.
